# Response-Related Potentials during Semantic Priming: The Effect of a Speeded Button Response Task on ERPs

**DOI:** 10.1371/journal.pone.0087650

**Published:** 2014-02-06

**Authors:** Marijn van Vliet, Nikolay V. Manyakov, Gert Storms, Wim Fias, Jan R. Wiersema, Marc M. Van Hulle

**Affiliations:** 1 Laboratory for Neurophysiology, KU Leuven, Leuven, Belgium; 2 Department of Experimental Psychology, KU Leuven, Leuven, Belgium; 3 Department of Experimental Psychology, Ghent University, Gent, Belgium; 4 Department of Experimental Clinical and Health Psychology, Ghent University, Ghent, Belgium; University of Barcelona, Spain

## Abstract

This study examines the influence of a button response task on the event-related potential (ERP) in a semantic priming experiment. Of particular interest is the N400 component. In many semantic priming studies, subjects are asked to respond to a stimulus as fast and accurately as possible by pressing a button. Response time (RT) is recorded in parallel with an electroencephalogram (EEG) for ERP analysis. In this case, the response occurs in the time window used for ERP analysis and response-related components may overlap with stimulus-locked ones such as the N400. This has led to a recommendation against such a design, although the issue has not been explored in depth. Since studies keep being published that disregard this issue, a more detailed examination of influence of response-related potentials on the ERP is needed. Two experiments were performed in which subjects pressed one of two buttons with their dominant hand in response to word-pairs with varying association strength (AS), indicating a personal judgement of association between the two words. In the first experiment, subjects were instructed to respond as fast and accurately as possible. In the second experiment, subjects delayed their button response to enforce a one second interval between the onset of the target word and the button response. Results show that in the first experiment a P3 component and motor-related potentials (MRPs) overlap with the N400 component, which can cause a misinterpretation of the latter. In order to study the N400 component, the button response should be delayed to avoid contamination of the ERP with response-related components.

## Introduction

Semantic priming refers to the case where the presentation of a prime stimulus affects the response to a later target stimulus [1]. When the prime stimulus is related to the target (for example associatively or semantically [2]), the target is processed more efficiently. An example would be a task where the subject is reading word-pairs, where each word flashes sequentially on a screen. Behavioral responses to the word *dog* will be faster when preceded by the word *cat*, compared to the word *sock*. This increase in efficiency is attributed to our semantic memory [1], [3]. To demonstrate the priming effect one can measure the response time (RT) on a task that requires the subject to process the stimuli [4]. It can also be measured with electroencephalography (EEG), where it manifests as an event-related potential (ERP) component called the N400 [5], [6].

Many semantic priming studies use a task that requires the subject to look for some property of the stimulus currently presented [7]. For example, a commonly used task is lexical decision [8], in which the subject is asked to make a decision about whether the presented string of letters is a valid word or a non-word. The subject presses one of two buttons as quickly as possible to give a response. The difference in RT is analyzed between valid word strings that were preceded by a related stimulus and valid word strings that were preceded by an unrelated one. Since such a task is known to generate a P3 component of which the latency correlates with the RT rather than the onset of the target stimulus [9], [10], we classify the component as response-related as opposed to stimulus-locked. Response-related components can be visualized by cutting EEG segments locked to response onsets and average them to get a response-locked ERP [11].

When conducting a semantic priming experiment designed to study the N400 component, Kutas et al. [12], [13], Duncan et al. [14] and Picton et al. [7] discourage the use of a button press in the time window used to analyze the stimulus-locked ERP, because a P3 component may be generated that overlaps with the N400 [15]. This recommendation is made as a side note, but deserves more attention as studies, that use a task where the subject presses a button during the ERP time window, continue to be published. For example, studying the ‘Method’ sections of the results of a search using Scirus(www.scirus.com), with the query “N400” “reaction time” “response time” where the results were limited to journal articles published in 2011 alone, yielded 7 semantic studies that mixed recording RT on a button press with ERP analysis. Two of them analyze both the stimulus-locked and response-locked ERPs, while the rest disregard response-related effects on the ERP completely.

Because of this, we feel a study dedicated to the problem is in order to examine the generated components in more detail. In this study, we try to gauge the severity of the distortion and the implications for the resulting conclusions drawn from such data. For this purpose, two experiments were performed: one where the subjects performed a speeded button response task and one where the response was delayed. The generated ERPs are analyzed to demonstrate the risk of contaminating stimulus-locked potentials, such as the N400, with response-related ones, such as the previously mentioned P3.

## Materials and Methods

In a semantic priming study across two experiments, subjects read a series of sequentially presented words, organized in pairs. In the first experiment the subjects pressed one of two buttons to indicate whether the two words of a word-pair were related or not as quickly as possible while remaining accurate in their decision making. In the second experiment the subjects performed the same task but delayed their button response until a cue was given. The two experiments will be referred to as the ‘speeded condition’ and ‘delayed condition’ respectively.

### Subjects

The experiment employing the speeded condition was performed with 10 university students (3 female, aged 19–27 years), all right-handed and native speakers of Flemish-Dutch. As the main interest of this study is the effect of delaying the subject’s button response, the experiment employing the delayed condition was performed with the same subjects from the first experiment to reduce between-subject variability. Because the recordings of the speeded condition were already completed before the inception of this study and the construction of the delayed setting, all subjects performed the speeded task first, followed by the delayed task at a later time. Since memory effects influence the N400 potential [16], subjects performed the latter experiment a minimum of 2 months after the first to mitigate these effects [17].

### Ethics Statement

This study was approved by the UZ Leuven ethics committee. All subjects were volunteers and signed an informed consent form before each experiment.

### Materials

To construct word-pairs with varying associative relatedness, an association norm dataset, compiled by De Deyne and Storms [18], [19], was used.

**Figure 1:**
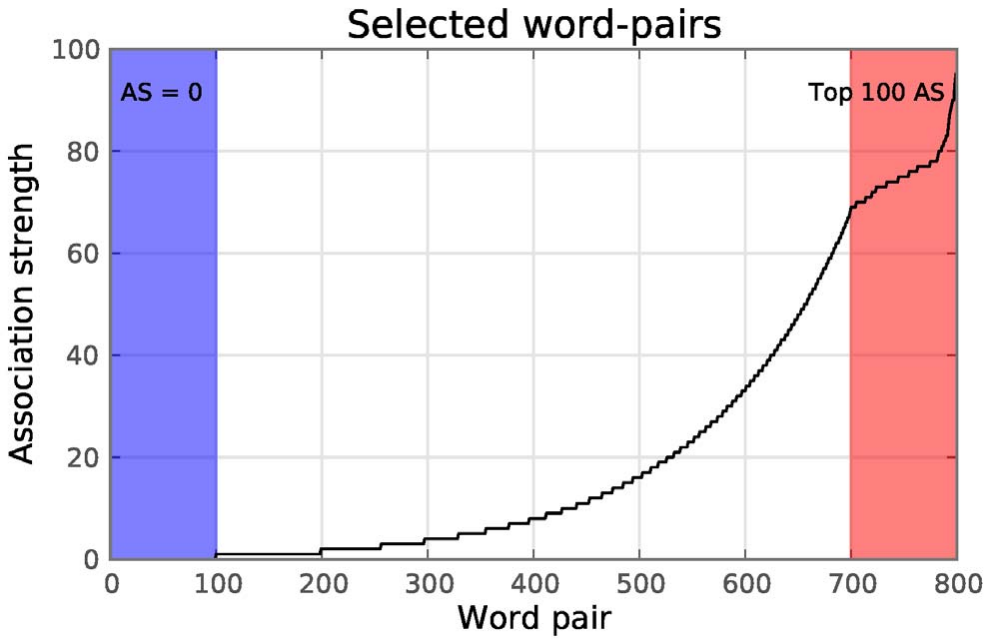
Association strength (AS) of all 800 word-pairs that were used. The blue shaded area contains 100 word-pairs with an AS of zero, meaning the words are completely unrelated. The red shaded area contains the 100 word-pairs with the highest AS in the association norm data. Both words are four to six letters in length. The AS of the remaining word-pairs follows a log scale.

The stimulus list used during the experiments consists of a total of 800 Flemish-Dutch word-pairs, selected (Fig. 1) with varying association strength (AS) from the association norm dataset mentioned above. AS was determined through a free association task, where cue words were presented to 100 subjects. They wrote down the first three words that came to mind to each cue [18], [19]. The AS of a (prime, target) word-pair is defined as the number of subjects that wrote down the target word in response to the prime word. In this study, only the first association of each subject is considered. The stimulus list consists of the top 100 strongest related word-pairs (AS ranged 69–95, mean AS = 75.62) and 100 word-pairs where the prime and target words were randomly chosen and no record of the word-pair existed in the association norm data, therefore having an assumed AS of 0. The remaining 600 word-pairs were chosen such that the logarithm of their AS score is uniformly distributed on the range [0… 69], extending the complete range between the unrelated and the top 100 strongest related word-pairs. The log scale was chosen because when the association norm data were analyzed, some properties of the word-pairs that co-vary with the AS, correlate better with its logarithm than the raw values. For example, the length of the target word (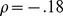 and 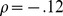 respectively) and the in-degree of the word-pair (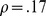 and 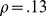 respectively). A word’s in-degree is the number of unique words to which the participants in the free association study generated the target word. This is a measure of the centrality of the word if the norm dataset is visualized as a semantic network [18]. Based on these logarithmic relationships, we hypothesize that the relationship between RT/N400 and AS might also be logarithmic. This hypothesis is tested in the results section.

All selected words for the stimulus list have a length of 4–6 letters and only reasonably common words were chosen. This was achieved by limiting the minimum word frequency to 2 occurrences per 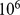 words in the SUBTLEX-NL corpus [20]. Also, the second word of a word-pair (i.e. the target word) had a minimum in-degree of 5, meaning they were well connected in the association norm data, which is an indication that they should be familiar.

In addition to capturing the button response of the participant, EEG was recorded continuously using 32 active electrodes (extended 10-20 system) with a BioSemi Active II System (BioSemi, Amsterdam, the Netherlands), having a 5th order frequency filter with a pass band from 0.16 Hz to 100 Hz, and sampled at 2048 Hz. An electro-oculogram (EOG) was recorded as recommended by Croft et al. [21]. Two electrodes were placed on both mastoids and their average was used as a reference for the EEG.

Stimulus presentation was done using MATLAB (MathWorks, Natick, Massachusetts, U.S.A.) and Psychtoolbox [22]. EEG data was processed using the NumPy/SciPy Python packages [23]. Figures were created using the Matplotlib Python package [24]. Linear mixed effect (LME) models were fitted using the LME4 package for R [25].

### Experimental Procedure

Subjects were seated in an upright position approximately one meter from a computer screen. The dominant hand rested upon the table with the index and middle fingers resting on mouse buttons.

A trial consisted of the sequential presentation of a single word-pair. The stimuli were shown as white text on a black background in the Arial font with a point size of 50 and centered on the screen both horizontally and vertically. The subject was instructed to press the left mouse button to indicate the prime and target were related or the right mouse button to indicate they were not. This can be seen as a simplified version of the judgment of associative memory (JAM) task [26]. Responses were performed by the index and middle fingers of the dominant hand, which was the right for all subjects. The mapping of the response to the mouse buttons and the hand used for responding were not counterbalanced. Normally, the hand used to respond and the mapping of the mouse buttons to ‘related’ and ‘unrelated’ responses is counterbalanced across recordings. This reduces differences due to left/right lateralized effects in the grand average RTs and ERPs. This study however analyzes potentials generated by the response of the subjects by averaging response-related components across subjects. Therefore, we tried to reduce the variability between the responses as much as possible by making all subjects respond with their dominant hand (for all subjects, the right hand) at all times and did not counterbalance the mapping of the mouse buttons. It is possible that due to the lack of counterbalancing, the spatial location of ERP components such as the N400 and P3 influenced by this fixed response button assignment.

In each experiment, 20 trials using word-pairs that are not part of the stimulus list, were presented for the subject to practice. Next, 800 trials, split up into 5 blocks of 160, were presented. Between each block the subject was prompted to take a short break. Each experiment lasted between 35 and 45 minutes, depending on the length of the breaks.

In the speeded condition, during each trial, the prime word was presented for 200 ms and the target-word for 1000 ms with a stimulus onset asynchrony (SOA) of 500 ms. Between trials, a blank screen was presented for one second. In the speeded condition, the subjects were told to respond as quickly and accurately as possible upon being presented with the target word. The delayed condition employed the same procedure with the exception that the target word would stay on the screen for 1500 ms, turning from white to yellow after 1000 ms. Subjects were told to delay their response until after the target word had changed color. In both conditions, the subjects had 1000 ms to respond or a no-response code would be logged instead.

### Data Preprocessing and Extracting Trials

The EEG was bandpass filtered offline between 0.1–50 Hz by a 3rd order two-way IIR filter to attenuate large drifts and irrelevant high frequency noise, but retain eye movement artifacts. It was downsampled to 256 Hz afterwards. The EOG was used to attenuate eye artifacts from the EEG signal using the regression method outlined in [21]. As we are mostly interested in examining N400, P3 and motor related (MRP) components, the EEG signal was band pass filtered again, between 0.5–15 Hz by a 3rd order two-way IIR filter, to further attenuate signals that are not of interest in this study. After frequency filtering, the signal was cut into segments from 0.1s before the onset of the target stimulus to 1.5s after. Baseline correction was performed using the average voltage in the 0.1s interval before the stimulus onset as baseline value. Finally, trials in which the subject had not made a button response, or was too late in making a response, were discarded.

### Statistical Analysis

Statistical analysis of various effects were done by means of a linear mixed effects (LME) model. For all usages of LME models in this article, random effects consisted of subjects (modeling both slopes and intercepts) and word-pairs (modeling intercepts only). Models were fitted using Restricted Maximum Likelihood (REML). Because the degrees of freedom in an LME model are non-trivial, 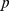-values were estimated using a Markov Chain Monte Carlo method. This design follows the recommendations of Baayen et al. [27].

When testing for a correlation between two vectors **x** and **y**, **x** was entered as the dependent variable in the LME model and **y** as a fixed effect (subjects and word-pairs were random effects). When testing for a difference between the means of two groups **x**_1_ and **x**_2_, we concatenated the values into a single vector *x* and used a coding vector **y** to label each value 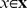 with a corresponding 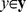, where 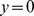 if 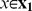 and 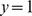 if 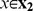. In the LME model, **x** was entered as the dependent variable and **y** as a fixed effect (subjects and word-pairs were random effects). In both cases, we report the obtained regression weight (*w*), the *t*-value (*t*) and *p*-value (*p*).

## Results

For both conditions 8000 trials (10 subjects × 800 trials) were initially collected. Rejection of no-response trials brought this number down to 7759 (3.0% rejected) EEG sweeps for the speeded and 7949 (0.6% rejected) for the delayed condition. For each subject, ERPs were constructed by sorting the trials by either the AS of the stimulus or the RT, grouping them into 8 non-overlapping bins of equal size (Table 1) and averaging the trials in each. Finally, the ERP of each bin was averaged across subjects to form the grand-average stimulus-locked ERPs and response-locked ERPs.

**Table 1:** Descriptive statistics of the bins across all conditions.

	Speeded condition, sorted by AS			Speeded condition, sorted by RT			Delayed condition, sorted by AS		
bin	mean AS (std)	mean RT (std)	resp	mean AS (std)	mean RT (std)	resp	mean AS (std)	mean RT (std)	resp
1	76.05 (5.27)	0.49 (0.122)	0.98	35.86 (30.49)	0.37 (0.035)	0.99	75.93 (5.29)	1.26 (0.131)	0.98
2	49.51 (9.98)	0.53 (0.127)	0.97	30.85 (28.31)	0.44 (0.015)	0.99	48.60 (10.01)	1.27 (0.131)	0.97
3	24.50 (5.03)	0.54 (0.131)	0.94	27.01 (27.27)	0.49 (0.013)	0.96	23.76 (4.93)	1.26 (0.129)	0.95
4	11.95 (2.52)	0.57 (0.138)	0.92	22.41 (25.74)	0.53 (0.013)	0.91	11.52 (2.44)	1.26 (0.133)	0.94
5	05.66 (1.29)	0.58 (0.139)	0.90	17.94 (23.42)	0.58 (0.015)	0.82	05.46 (1.22)	1.26 (0.132)	0.90
6	02.57 (0.63)	0.60 (0.148)	0.82	13.76 (20.93)	0.64 (0.019)	0.68	02.48 (0.57)	1.27 (0.130)	0.85
7	01.06 (0.23)	0.62 (0.146)	0.72	13.65 (21.96)	0.71 (0.026)	0.61	01.02 (0.15)	1.27 (0.144)	0.71
8	00.03 (0.17)	0.67 (0.145)	0.26	09.69 (17.40)	0.85 (0.059)	0.54	00.01 (0.11)	1.26 (0.134)	0.21

For each condition, the mean and standard deviation of AS and RT are listed, as well as the response ratio of each bin. The response ratio is the number of ‘related’ JAM responses given by the subjects, divided by the total number of responses. The conditions correspond to Fig. 3A, 3B and 3C respectively. Units for RT are seconds.

### Button Responses

In the speeded condition, RT shows an inverse dependency to the AS of the word-pair (Table 1). Statistical analysis of the effect was done by means of an LME model with RT as the dependent variable and AS as a fixed effect (subjects and word-pairs were random effects). Two models were constructed: one that used the raw AS values and another that used the logarithm of the AS (dropping the trials in which AS was zero). The model using the log AS provided a better fit on the data (log likelihood -10098) than the model using the raw AS values (log likelihood -10166). For the speeded condition, the model indicated a significant effect of log AS on RT (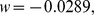 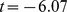, 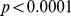). No effect was found for the delayed condition (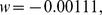 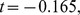 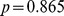). It is likely the subject had already prepared his/her decision whether the two stimuli are related in advance.

**Figure 2:**
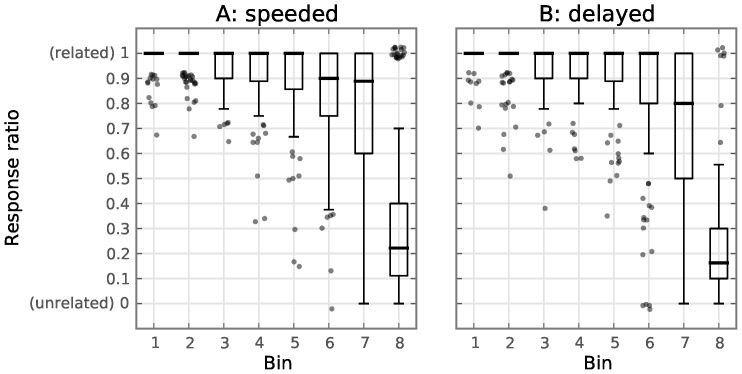
Boxplots of button responses made by the subjects. Each boxplot corresponds to a bin (see table 1). Whiskers extend to the inner quartile range. Outliers are plotted as semi-transparent dots, with some random jitter applied to the x (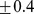) and y (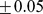) position to reduce overlap. Bin 1 contains trials with a high AS and subsequent bins contain trials with lower AS. Bin 8 contains mostly trials with 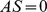 (the trials in this bin with 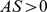 are mostly outliers in the corresponding boxplot). The y-axis shows the number of subjects that rated the two words of the word-pair as being related, divided by the total number of subjects (10). **A**: responses during the speeded condition. **B**: responses during the delayed condition.

During the experiment, each word-pair was rated by the 10 subjects by either pressing the left (unrelated) or right (related) mouse button. We must point out that the purpose of the response task was to keep the subjects focused during the experiment, rather than obtaining reliable JAM ratings. No corrections were for example performed for response bias due to the order of the stimuli (after a long series of unrelated word-pairs, a subject would be biased toward rating a new word-pair as related). We refer to the number of times a ‘related’ JAM response was given, divided by the total number of responses, as the ‘response ratio’. Table 1 shows the response ratio for each bin and it can be seen that as the AS between words increases, the likelyhood of a ‘related’ JAM response increases as well. A break from the overall trend occurs between word-pairs with 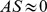 (bin 8, sorted by AS) and 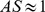 (bin 7, sorted by AS) as the response ratio drops sharply. This contributes some evidence that a log scale is suitable for AS, as 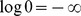 and 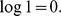 Fig. 2 shows the distribution of the response ratios of the word-pairs in each bin, where the response ratio corresponds to the number of subjects that gave a ‘related’ JAM response to the word-pair, divided by the number of subjects (10). These response ratios show a pattern, which is similar during both the speeded and the delayed conditions 2. At low AS levels, instead of all subjects agreeing that the words are unrelated, a high variance is seen. Indeed, a good portion of the word-pairs with AS = 1 were unanimously rated as related by all subjects. As also shown in a study by Maki [26], subject’s JAM ratings are generally higher than the free association scores.

### ERPs During the Speeded Condition

**Figure 3:**
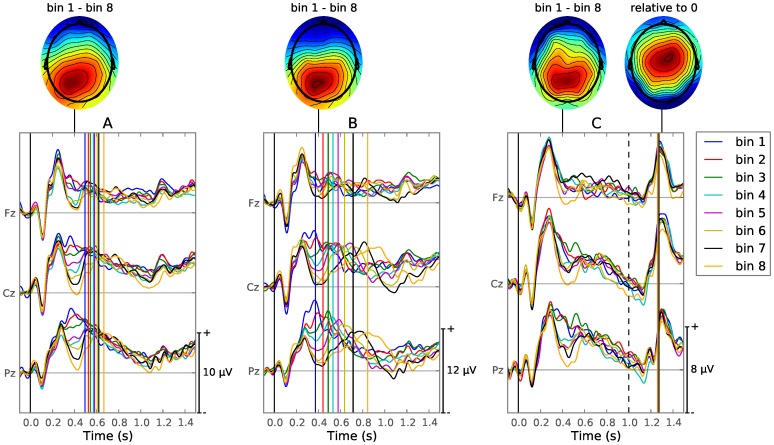
Stimulus-locked ERPs and scalp topographies for both experimental conditions. Vertical lines are plotted to show the mean RT of the trials belonging to each bin (Table 1), using the same color code as the ERPs. Scalp topographies are drawn for components of interest, using red for positive and blue for negative values. Note that the y-axes have different scales and each scalp topography uses its own normalized scale. **A**: Stimulus-locked ERP of the speeded condition; trials sorted by decreasing AS. The scalp topography shows the difference between bins 1 and 8 at 400 ms. **B**: Stimulus-locked ERP of the speeded condition; trials sorted by increasing RT. Scalp topography shows the difference between bins 1 and 8 at 400 ms. **C**: Stimulus-locked ERP of the delayed condition; trials sorted by decreasing AS. A vertical dotted line indicates the moment the target word turned yellow, which cued the response of the subject. Two scalp topographies are drawn, one showing the difference between bins 1 and 8 at [400]ms, the other showing the voltage at 1300 ms relative to zero.

The ERPs recorded during the speeded condition suggest a strong N400, which becomes more negative as the AS between the words becomes smaller (Fig. 3A). The timing and scalp topography of this effect is very similar to the one described in the literature ([6], Fig. 1). Statistical analysis was performed on the difference between the first and last bins. For each individual EEG sweep belonging to either the first or the last bin, the voltage at electrode Pz over the time-range 300–500 ms was used to quantify the candidate N400 component. An LME model was constructed to test the difference of the average EEG voltage between the first and last bins (see the methods section for details), which was found to be significant (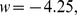 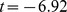, 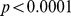).

**Figure 4:**
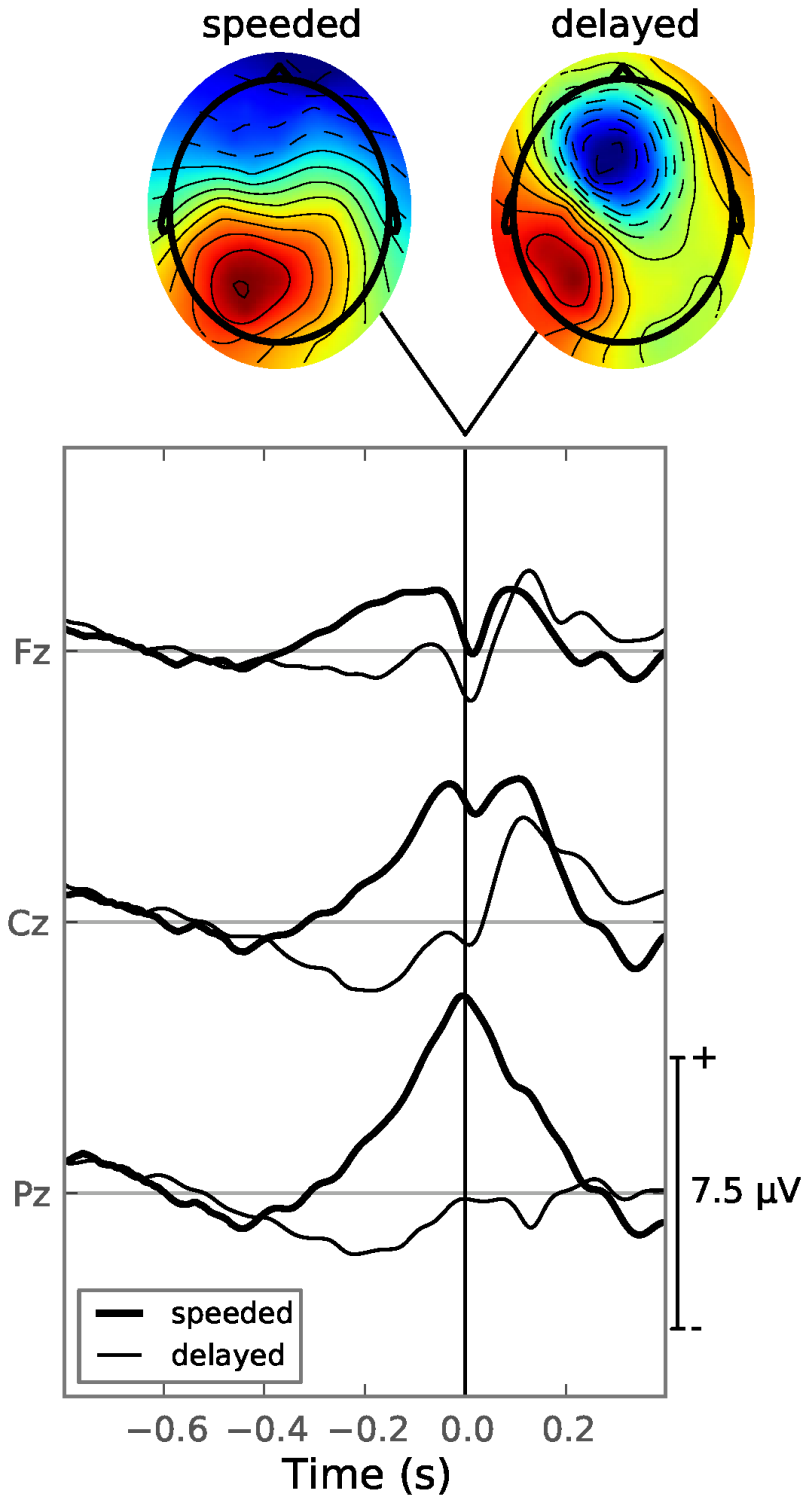
Response-locked ERPs and scalp topographies for both experimental conditions. Shown are the grand response-locked ERPs across all bins and all subjects for the speeded condition (thick line) and delayed condition (thin line). Scalp topographies are given showing the voltage at 0 s, using red for positive and blue for negative values. Note that both scalp topographies use their own normalized scale.

When comparing short versus long RTs, it becomes clear that more processes are going on in the same time window, as a large component is now seen aligned to the mean RT of the bin (Fig. 3B). A statistical analysis of the latency of this component, using a template matching technique, is given in a later section. This component is also present in the response-locked ERPs (Fig. 4, thick line). The topographies of the different components are very similar: posteriorly and slightly to the left. The latter most likely due to the fact that all subjects responded using their right (for all subject, the dominant) hand.

A similar component was described in various RT experiments [9], [28], and was in those studies classified as a P3. Kutas et al. (1977) observed that during a speeded task, the P3-latency and RT are strongly correlated, but not necessarily equal. This lead to the conclusion that the P3-latency could be a correlate of stimulus processing time. Since AS and RT are also correlated (as shown in the previous subsection), bins with a different mean AS will also have a different mean RT (Fig. 3A, vertical lines). It is likely that the effect seen in the AS-binned case consists of not only the N400, but is in fact dominated by the difference in latency of the P3 component seen in the RT-binned case, which has a similar scalp topography and overlaps in time with the N400.

### ERPs During the Delayed Condition

During the delayed condition (Fig. 3C), a component is seen which is very similar to the one described in the speeded condition. Namely it occurs at 400 ms after the onset of the target word and is increasingly negative as the AS of the bin decreases. Analyzing the difference in mean EEG voltage of electrode Pz in the time-range 300–500 ms, between the first and last bins, shows a significant difference (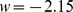, 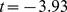, 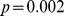). Additionally, a strong component can be seen at 1300 ms, 300 ms after the target word turned yellow, which cued the button response of the subject. Although the mean latency of this component is close to the mean RT (Fig. 3C, vertical lines), it is not visible in the response-locked ERP (Fig. 4, thin line). Judging from the timing and scalp topography of this component, it’s likely a P2 generated by the response cue [29]. Similar P2s can be seen around 300 ms after the onset of the word stimulus during both the speeded and delayed conditions. The response-locked potentials for the delayed condition contain mostly motor related potentials (MRP) with a similar shape as described in the literature [30], [31]: a negative slope (NS) leading up to a negative, mostly anterior, motor potential (MP) at the moment of the button press (Fig. 4, thin line).

The components generated during the delayed condition (Fig. 3C and Fig. 4, thin line) are spatially more clearly separated. The N400 occurs central-posterior, whereas the P2 component at 1300 ms occurs slightly anteriorly, slightly to the right. The MP (Fig. 4, delayed condition) displays a dipole pattern at the onset of the button press with the positive component at a left-posterior location and the negative component central-frontal.

### Analyzing P3-latencies

To further study the P3 component observed in the speeded task, we repeated the analysis done by Kutas et al. [9] and attempted to estimate its latency for each trial. For each subject, a slightly modified version of the template matching technique developed by C. D. Woody [32] was applied. Since this technique operates on one-dimensional data, we limited the analysis to the Pz electrode:

1. Filter the signal with a 3rd order, two-way, Butterworth filter between 0.5 and 6 Hz in order to remove alpha activity.
2. Take the RT for each trial as the initial P3-latency estimate.
3. Based on the P3-latency estimates, cut the EEG signal for each trial between -0.5 and +0.5s, relative to the latency estimate. Average the cuts to generate a ‘P3-locked’ ERP. This ERP becomes the new template.
4. For each trial: calculate the cross-correlation of the signal with the template. We restricted this analysis to the time window from 350 to 900 ms after the onset of the target stimulus. This yields a vector containing for every sample a score. Determine the peaks in the score vector by detecting sign changes in the derivative. Take the position of the largest positive peak to be the new estimated P3-latency.
5. Repeat steps 3–4 four times to refine the template.

**Figure 5:**
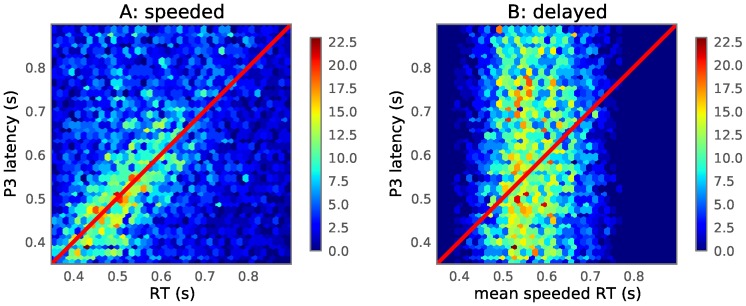
Two-dimensional histogram of single trial component latency versus RT. A hexagonal grid shows histograms of component latency versus RT for all non-rejected trials of all subjects. The values along the x and y axes indicate seconds since the onset of the target stimulus. Component latency was determined through iterative template matching (see the results section). **A**: P3 latency during the speeded condition; superimposed as a red line is 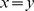. **B**: P3 latency during the delayed condition, versus the mean RT of each word-pair collected during the speeded condition.

For the speeded condition, it can be seen that, on average, the P3-latency follows the RT (Fig. 5A), explaining the alignment of the two in Fig. 3B. However, the P3 component is not strictly aligned on the response, sometimes occuring before or after the onset of the button press. This finding is consistent with Kutas et al. [9]. They postulated that the P3 is an index of the time it takes to process the stimulus and make a decision. Statistical analysis of the effect was done by using P3-latency as the dependent variable and RT as the fixed effect of the LME model. For the speeded condition, the model indicated a large effect of RT on P3-latency (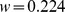, 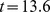, 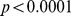). Repeating the analysis with the logarithm of AS as fixed variable also yielded a significant effect (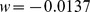, 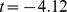, 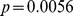), which comes as no surprise as the RT was shown above to correlate strongly with AS. However, the model using RT as a fixed effect has a much better fit to the P3-latency data (log likelyhood 3855) than the model using AS (log likelyhood 3781 ). Presumably, this is because semantic priming is influenced by many factors besides AS, such as word length and frequency. These factors are all reflected in the RT of the subject.

There is a possibility that a P3 is also generated in the delayed condition during the time leading up to the response cue as the subject makes up his/her mind. We employed the template matching technique to find P3-latencies in the 350 to 900 ms window after the onset of the target stimulus. However, since in the delayed condition, RTs cannot be used as the initial P3-latency estimate in step 2, since RTs generated during this condition are all outside of the 350–900 ms time window. Instead, the final template used to estimate the P3-latency during the speeded condition was re-used as initial template for the delayed condition. The obtained P3-latencies were analyzed with an LME model. No significant effect was found using AS as a fixed effect (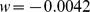, 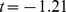, 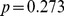). We observed earlier that, when using a speeded task, RT was a better predictor of P3-latency than AS. For each word-pair, we calculated the mean RT, obtained during the speeded condition, and used it as a predictor for the P3-latency during the delayed condition (Fig. 5B). This ‘mean speeded RT’ predictor variable does show a very small, but significant effect when used as fixed effect in the statistical model (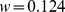, 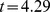, 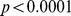), despite being hardly noticable in Fig. 5B. This still leaves open the question whether during the delayed condition, a P3 is generated which latency correlates with the AS of the word-pair, since the effect is too small to be reliably detected by our template matching technique, which was successful in detecting the P3 generated during the speeded condition.

### Impact of P3-latency on Overall ERP

We noted before that the P3 potential observed in the speeded condition overlaps in time and scalp topography with the N400 as described in the literature. To demonstrate how a difference in P3-latency can mask N400 effects, four groups of trials were made in such a way that N400 and P3 effects are in competition. The first group consists of trials recorded during the speeded condition, where the stimulus AS was low (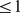) and the P3-latency was short (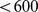
ms). The second group consists of trials also recorded during the speeded condition, where the stimulus AS was high (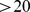) and the P3-latency was long (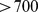
ms). Note that we would expect the first group to have a more negative N400 than the second group (based on AS), but also a shorter P3-latency (based on the latency estimates). Equal group sizes were enforced by randomly discarding trials from the larger group. The third and fourth groups consisted of the trials recorded during the delayed condition that correspond in terms of subject and stimulus to the trials in the first and second groups. In case a subject-stimulus combination was not available due to rejection of no-response trials, it was removed from all groups. In the end, all groups contained 635 trials. The P3-latency of the chosen trials are unusual, because normally a trial with a high AS stimulus would result in a short P3-latency. The response ratios for the four groups are 0.52, 0.96, 0.97 and 0.97 respectively. The AS of the trials in groups 1 and 3 would place them in bins 7–8 (sorted by AS) in Table 1, which have an average response ratio of 
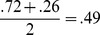
 in the speeded and 
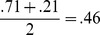
 in the delayed condition. The AS of the trials in groups 2 and 4 would place them in bins 1–3, which have an average response ratio of 
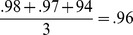
 in the speeded and 
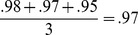
 in the delayed condition. The response ratios of the trials in the four groups are representative of those of the entire dataset, even if the P3-latencies are unusual.

**Figure 6:**
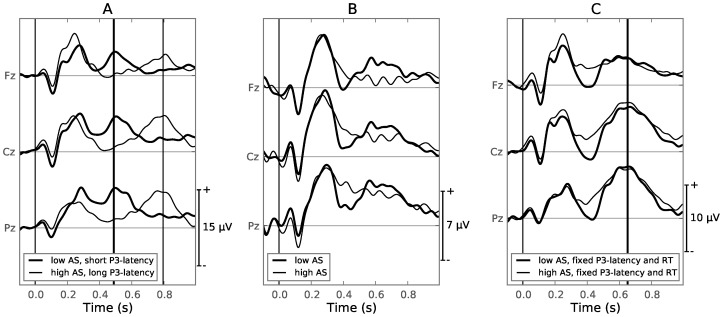
Grand average ERPs demonstrating the effect of differences in P3-latency. A : Speeded condition, low AS (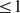) and short P3-latency (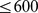 ms, thick line) versus high AS (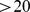) and long P3-latency (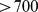 ms, thin line). Vertical lines indicate the mean P3-latency of each group. **B**: Delayed condition, using the same subject/word-pair combinations used to create A. **C**: Speeded condition, low AS (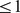, thick line) versus high AS (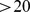, thin line) using a fixed P3-latency and RT (around 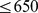 ms). Vertical lines indicate the mean P3-latency of each group, which overlap in this case.

Comparing the ERPs of the four groups shows that the P3 component dominates the ERP in the speeded condition (Fig. 6A). While the low AS group portrays a distinctive negative peak at 400 ms, the P3 occurs shortly afterwards, causing a net positive difference with the high AS group. In other terms, the P3-latency effect ‘wins’ over the N400 effect. Analyzing the mean voltage between 400 and 500 ms for electrode Pz, yields a significant difference between the two groups (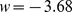, 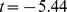, 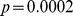). This would us to to conclude that the target-words of the first group were processed faster than the target-words of the second. However, we must be careful not to attribute this to a property inherent to the word-pairs, such as declaring that for these word-pairs the subjects disagree with the association norm data. When we look at the delayed condition (Fig. 6B), using matched subjects and matched stimuli, the N400 component is no longer visibly obscured by the P3. Based on the N400 amplitudes of both groups, we would now draw the opposite conclusion in this case (comparing the mean voltages between 400 and 500 ms of electrode Pz in a similar fashion as before: 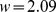, 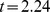, 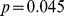).

Finally, two more groups of trials were created using trials recorded during the speeded condition: a low-AS (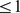) and a high-AS (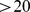) group, where all trials have a similar P3-latency and RT. This should let us compare the difference in N400 amplitude between the low-AS and high-AS cases, without distortion due to differences in P3-latency. Consider the following scoring function:
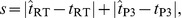
where 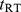 is the RT of the trial and 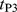 is the estimated P3-latency of the trial. Low values of 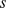 correspond to trials with RT close to 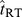 and P3-latency close to 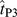. To construct the low-AS group, for each subject, out of all trials with 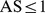, the 75 trials with the lowest score 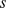 were selected. In the scoring function, both 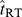 and 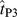 were set to 0.65s to avoid overlap of the P3 component with the N400. The high-AS group consists of trials that correspond to the trials in the low-AS group in terms of subject, RT and estimated P3-latency. For each trial in the low-AS group, the scoring function, with 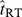 set to the RT of the low-AS trial and 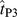 set to its P3, was used to score all trials of the same subject and with 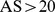. The trial with the lowest score was selected. The end result were two groups of 750 trials with an equal mean P3-latency (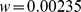, 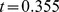, 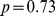) and mean RT (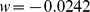, 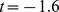, 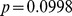). Comparing the ERPs of both groups (Fig. 6C) shows the N400 potential during the speeded condition without interference of the P3 (comparing the mean voltages between 400 and 500 ms of electrode Pz: 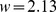, 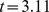, 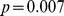).

## Discussion

During the experiments, the subjects were asked to read a word-pair, rate it either as related or unrelated and press the corresponding button. Based on the stimulus- and response-locked ERPs, we can identify at least four sources of ERP components. When a stimulus is displayed on the screen, a series of components, among which a strong P2 component, is evoked by it. Next, the experiments were designed to evoke a priming effect that is known to generate an N400, so this component is expected to be present in both speeded and delayed conditions. This component is linked to the semantic processing of the words [6]. Additionally, the subject is required to choose which button to press. In the speeded condition this occurs as soon as the subject has decided whether the words are related and the decision generated a P3 component close to the moment when the button was pressed. This causes the P3 to be best visible in the response-locked ERP. In the delayed condition, during the one second interval between the onset of the target word and the onset of the response cue, the subject has time to decide whether the word-pair is related or not, so one might expect a P3 component to occur. However, the template matching technique that was successful in showing a positive relationship between P3-latency and AS during the speeded condition failed to do so during the delayed condition. Finally, when the subject presses the button, response-locked MRPs are generated leading up to, as well as occurring at, the onset of the button press [31].

In the speeded condition, the N400, P3 and MRP components have overlapping time windows, leading to a mixture that presents itself in the stimulus-locked ERP, in the low-AS versus the high-AS case, as a difference wave very similar to the N400 component alone (Fig. 3A). Even if the N400 would not be generated at all, the combination of the P3 and MRP added together forms a mixture (Fig. 4, thick line) which overlaps in time and topography with that of the N400 component. This makes it very difficult to accurately assess the magnitude of the N400 present in the EEG signal, leading to conclusions that are more likely based on the P3-latency than the N400, as demonstrated in Fig. 6A–B. To examine N400 effects alone, we demonstrated a template matching technique that can be employed to detect single trial P3-latencies, allowing a researcher to compare groups of trials, keeping both P3-latency and RT fixed (Fig. 6C).

## Conclusion

If the goal of the experiment is to capture N400 effects, we advise caution when the subjects perform a button response close to the time window of interest for ERP analysis. We presented evidence that large P3 and MRP components overlap with the N400, which causes difficulties isolating the latter. These findings justify the advise of Picton et al. [7], Kutas et al. [12], [13] and Duncan et al. [14] to not employ a response task in the same time window used when analyzing the N400. Where they merely advise against it without elaborating on the subject, our study demonstrates the severity of the issue. To study the N400 it is recommended that the subject is given an explicit task to keep him/her alert [7]. We recommend a design where the button response is delayed to avoid contamination of the ERP with response-locked components.

When a study requires RT data, and RT data cannot be acquired during a separate recording, one has to deal with the P3 and MRP components in some way. We demonstrated a simple template matching technique, developed by Woody [32], to estimate single trial P3-latencies. N400 effects can then be gauged by comparing groups of trials with equal mean P3-latency and RT. We encourage the reader to also look into spatial decomposition techniques such as independent-component analysis (ICA), which was employed successfully by Jung et al. [33], and temporal decomposition techniques, such as the one proposed by Takeda et al. [11], to separate response-locked and stimulus-locked components. A thorough discussion of these techniques is beyond the scope of this paper.

The argument can be made that the main conclusions of many linguistic studies are about whether a priming effect occurs in a certain condition or not. In this case it does not matter what components dominate the ERP, as long as a priming effect is demonstrated. While this reasoning is correct, we counter that in this case one can suffice with reaction time recordings only. Often the purpose of jointly recording reaction time and EEG is to gather *additional* information about the semantic processes in our brains. In this case one must be aware of the different components that are in play and not for example mistake a difference in P3-latency for an N400 component.
